# Dual regulation of TERT activity through transcription and splicing by ΔNP63α 

**DOI:** 10.18632/aging.100003

**Published:** 2008-12-09

**Authors:** Esther Vorovich, Edward A. Ratovitski

**Affiliations:** Department of Dermatology, The Johns Hopkins University School of Medicine, Baltimore, MD 21231, USA

**Keywords:** P63, p53, SIRT1, Sp1, TERT, mouse, transcription, splicing, senescence, ageing

## Abstract

P53 homolog p63 was shown to play a role in premature ageing phenotype found in mouse models through regulation of the replicative senescence.  We previously showed that the forced ΔNp63α expression decreased the SIRT1 protein levels, and induced the replicative senescence of human keratinocytes, while the ectopic SIRT1 expression decreased the senescence. Using the ΔNp63α overexpressing and *p63-/+* heterozygous mice, we found that ΔNp63α induced the mTERT promoter activation through the down regulation of the SIRT1 protein levels, inactivation of p53 deacetylation, decrease of the p53/Sp1 protein-protein interaction, and the overall induction of mTERT transcription regulation. In the same time, by a forming of protein-protein complexes with the ABBP1, ΔNp63α induced the mTERT RNA splicing leading to an increasing expression of spliced mTERT isoforms playing a role of dominant-negative inhibitors of mTERT activity and therefore decreasing the levels of TERT activity in mouse epidermal keratinocytes. The overall effect of the ΔNp63α overexpression resulted in decrease in telomerase activity and increase in replicative senescence observed in mouse keratinocytes.  This dual molecular mechanism of telomerase regulation might underline the previously shown effect of ΔNp63α on premature ageing phenotype.

## Introduction

Cell senescence and stress modulate the proliferative potential of mammalian cells, suggesting that both are capable of suppressing the formation of tumors [1-7].  Stresses and dysfunction of the telomeric DNA/telomerase complex can trigger senescence. Impaired telomere function activates the canonical DNA damage response pathway that engages p53 to initiate apoptosis or replicative senescence, while the inactivation of the tumor suppressor genes (*Rb* and *p53*) allowing cells to escape senescence [8-16]. The resulting cell immortalization is an essential component of the tumorigenic phenotype of human cancer cells [8-12,17].

Skin epidermis is one of the few regenerative tissues that express telomerase, the ribonucleoprotein complex that can counteract telomere erosion, one of the presently mostly favored potential mechanisms causing cellular ageing [18]. Altered functioning of both telomerase and telomere-interacting proteins is present in some human premature ageing syndromes and in cancer, and recent findings indicate that alterations that affect telomeres at the level of chromatin structure might also have a role in human disease [18-24].

P53 transcriptional factors are involved in regulation of cellular senescence and organismal ageing [14,16,25-30].  While p53 suppresses the onset of malignancy and, thereby extends lifespan, it induces cellular senescence and apoptosis upon DNA damage [8,9,14,16,25,26]. Transgenic mouse strains (p53+/m) expressing the C-terminal p53 fragment along with the wild type p53 display an early onset of phenotypes associated with ageing [30].  The ΔN-isoform of p53 recently reported [27,28] or ΔN-isoforms of p63 and p73 (all lacking the transactivation domain) might modulate an imbalance between them and full-length p53 leading to an altered transcriptional function of p53 and in turn to an acceleration of the ageing process [30,31].

Sirtuins possessing the histone deacetylase activity are implicated in the extension of lifespan of eukaryotic cells [29,32-40].  Epigenetic alterations of the expression of longevity genes by changing the level/pattern of histone acetylation may be an important factor in determining the longevity of animals [41,42].  SIRT1 encodes an NAD-dependent histone deacetylase that playing a critical role in transcriptional silencing [39,40].  Studies have implicated SIRT1 in binding to and deacetylating of the p53 protein (or Forkhead family members), inhibiting p53-dependent apoptosis, preventing a premature cellular senescence and leading to increase of organismal longevity [43,44].

We previously showed an important role for p53 homolog p63 (ΔNp63α), shown to be a key switch in skin renewal [25,45], in regulation of ageing process in p53+/m and ΔNp63α transgenic mouse models [29,30].  P63 was also shown to transcriptionally regulate many genes implicated in epithelial integrity, differentiation, and ageing [31].

## Results

### ΔNp63α induces the SIRT1 degradation and the p53/SIRT1 protein interaction

Mice overexpressing a truncated mutant of p53 (*p53+/m*, C-terminal part) or ΔNp63α were shown to exhibit a premature ageing of skin and shortened life span suggesting that these mice share a common molecular mechanism underlying these phenotypes [29,30].  The link between cellular senescence/premature ageing and p53 family members was reported by several groups [25,26,29,30].  *P63* deficiency was found to induce cellular senescence and to cause an accelerated ageing phenotype in adult mice showing the conditional expression or depletion in stratified epithelia contributed to ageing [29,30]. We have previously shown the expression of endogenous ΔNp63α in the *p53+/m* mice and overexpression of ΔNp63α in transgenic mice may play an important role in premature ageing [29]. We also found that the formation of ΔNp63α/SIRT1 complexes led to a decreased SIRT1 levels in both *ΔNp63α* transgenic and *p53+/m* mice [29].  We further observed that the marked senescence in the ΔNp63α overexpressing cells that could be modulated by a forced expression of SIRT1 [29].

**Figure 1.**
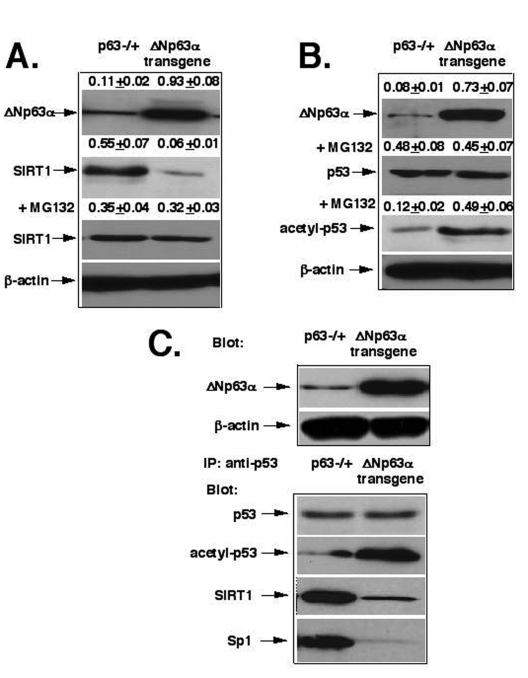
ΔNp63α mediates the SIRT1 degradation and p53 deacetylation. **(A)** The proteasome-dependent degradation of SIRT1. **(B)** The deacetylation of p53. **(C)** The protein complex formation between p53, SIRT1 and Sp1. Mice with heterozygous *p63-/+* and *ΔNp63α*transgenic expression were sources for epidermal keratinocytes [29,45].  Total lysates (2x10^5^ cells) were used for immunoblotting with indicated antibodies (dilutions: anti-ΔNp63, 1:500; anti-SIRT1, 1:300; anti-β-actin, 1:400; anti-p53, 1:500; anti-acetyl-p53, 1:400; anti-Sp1, 1:300).  Cells were also treated with the proteasome inhibitor, MG-132 (20 μg/ml) for 24 h before lysis. For immuno-precipitation (IP) experiments, we used total lysates obtained from 1x10^6^ cells/500 μl and anti-p53 antibodies (10 μg/500μl).  Blots were quantitatively scanned using the PhosphorImager and all of the data (mean +SD) were from at least three independent experiments.

For these studies, we used primary mouse epidermal keratinocytes obtained from mice with *p63-/+* heterozygous inactivation [45] and the *ΔNp63α* transgenic mice [29], as previously described [46,47].  Using the primary mouse epidermal cell culture, we found that the protein levels of SIRT1 were significantly lower (by 9-fold) in cells obtained from the *ΔNp63α* transgenic mice (0.06+0.01) than in the cells prepared from *p63-/+* mice (0.55+0.07, Fig. 1A).  We further found that the 26S proteasome inhibitor, MG-132, dramatically modulated the SIRT1 protein degradation effect, which was likely to be induced by ΔNp63α dramatically increasing the SIRT protein levels (Fig. 1A).  We also showed that levels of acetylated p53 were much greater (by 4- fold) in the *ΔNp63α* transgenic mice (0.49+0.06) than in *p63-/+* mice (0.12+0.02), while the p53 protein levels were practically unaffected (Fig. 1B). Next, we observed that the protein complex formation between p53, SIRT1 and Sp1 dramatically decreased in the *ΔNp63α* transgenic mice compared to *p63-/+* mice (Fig. 1D).

### ΔNp63α activates the transcription regulation of TERT core promoter

**Figure 2.**
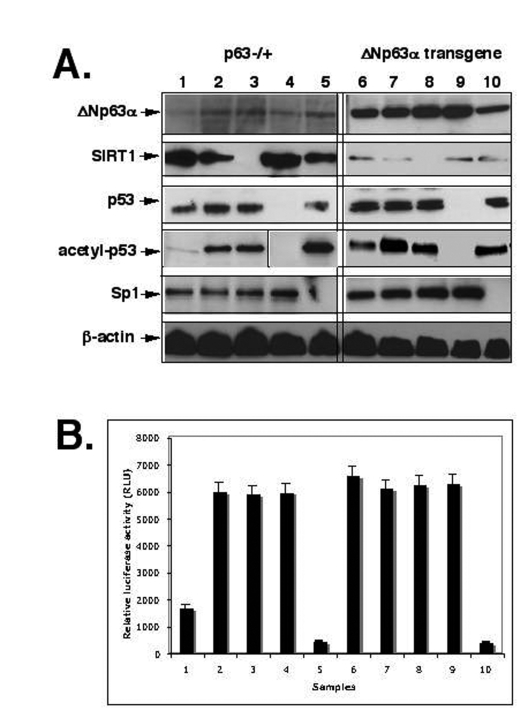
ShRNA silencing of ΔNp63-SIRT1-p53-Sp1 pathway. Mouse epidermal keratinocytes (2x10^5^ cells) from p63-/+ (samples 1-5) or overexpressing ΔNp63α(samples 6-10) were treated with control media (samples 1 and 6), SIRT1 inhibitor (Sirtinol, 100 μg/ml for 24 h; samples 2 and 7), or transfected with the SIRT1 shRNA (samples 3 and 8), p53 shRNA (samples 4 and 9), and sh-Sp1 RNA (samples 5 and 10). **(A)** Immunoblotting with indicated antibodies (dilutions: anti-ΔNp63, 1:500; anti-SIRT1, 1:300; anti-Sp1, 1:300; anti-p53, 1:500; anti-acetyl-p53, 1:400; anti-β-actin, 1:400). The vertical lines separate data obtained from independent protein gels. **(B)** mTERT promoter luciferase reporter assay. Mouse keratinocytes (1.0 x 10^5^) were transfected with the pGL3-347-Luc plasmid (0.5 μg) or the pGL3 control plasmid (0.5 μg) by using FuGENE6 transfection reagent (Roche Diagnostics). 3 ng of the pRL-SV40 (Promega) was used as a normalization control. Measurements were performed by using the Dual Luciferase reporter assay system (Promega) and a BioOrbit 1251 luminometer.  The activity of each TERT promoter fragment was expressed as a relative value. All of the data (mean +SD) were from at least three independent experiments.

The 3′-region of the core TERT promoter contains a GC-box, which binds Sp1 and is essential for transactivation and expression of the full-length telomerase [43,48-54]. Overexpression of Sp1 leads to a significant activation of transcription in a cell type-specific manner, while an interaction with p53 could eliminate the binding of Sp1, resulting in TERT repression [43]. To further examine this phenomenon, we used the inhibitor/RNA silencing approach to investigate the effect of the inhibition of SIRT1, p53 and Sp1 function on the transcriptional regulation of mouse telomerase-reverse transcriptase (mTERT) promoter. The epidermal cells form *p63-/+* mice and the *ΔNp63α* transgenic mice were transfected with shRNA for SIRT1, p53 and Sp1 or incubated with SIRT1 inhibitor, Sirtinol, as described elsewhere [36-38]. We, therefore, found that the SIRT1 expression led to a decrease of acetylated p53, while both Sirtinol and SIRT1 shRNA induced an increase of acetylated p53 (Fig. 2A). We further studied the effect of these treatments on luciferase reporter activity driven by Sp1 binding element of the mTERT promoter [53,54].  Mouse keratinocytes transfected with shRNA for SIRT1, p53 and Sp1 or treated with Sirtinol were also co-transfected with the murine core TERT promoter-Luc reporter vector (pGL3-347-Luc) containing the Sp1 binding site along with the Renilla luciferase plasmid as described elsewhere (Methods).  We showed that the overexpression of ΔNp63α results in a significant increase in transcriptional activity of the core mTERT promoter (Fig. 2B, samples 1 and 6).  We also observed that inhibition of SIRT1 expression or function, and p53 expression led to an increase of luciferase reporter activity, while silencing of Sp1 induced the down regulation of luciferase reporter activity (Fig. 2B).

**Figure 3.**
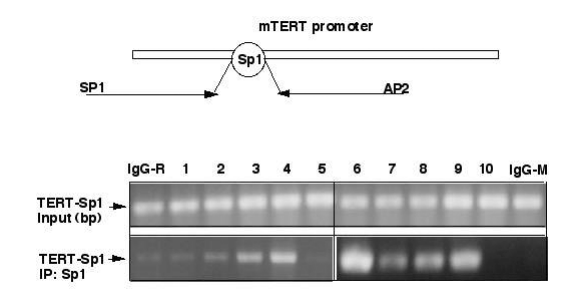
ΔNp63α modulates binding of Sp1 to Sp1 DNA-binding region by decreasing the SIRT1 protein levels and deacetylation of p53. Chromatin immunoprecipitation assay (X-ChIP). Mouse epidermal keratinocytes (5x10^7^ cells) expressing heterozygous p63-/+ (samples 1-5) and overexpressing ΔNp63α(samples 6-10) were treated with control media (samples 1 and 6), SIRT1 inhibitor (Sirtinol, 100 μg/ml for 24 h; samples 2 and 7), SIRT1 shRNA (samples 3 and 8), p53 shRNA (samples 4 and 9), and shSp1 RNA (samples 5 and 10). The protein-DNA complexes were precipitated with a primary antibody for Sp1. As negative controls, we used immunoglobulins (IgG) from rabbit (IgG-R) or mouse (IgG-M) sera. The mTERT-derived Sp1 promoter region using the following primers: sense (SP1), 5'-CTCACTGTCTGTGCAACCACAGCAGCTG-3' (position-363), and antisense (AP2), 5'-AGAGCACCGCGGGGCAACGAGGAGCGCG-3' (position +143) giving raise to a 506 bp PCR product. The PCR products were run on 2% agarose gels and visualized by ethidium bromide staining.

We then investigated whether the above-mentioned treatments affect endogenous transcriptional regulation of mTERT promoter using the chromatin immunoprecipitation (ChIP) approach using an antibody against Sp1 as described elsewhere [53,54].  We thus found that ΔNp63α overexpression induced an interaction of Sp1 transcription factor with the core promoter of mTERT (Fig. 3).  Similar effect was found in cells transfected with shRNA inhibiting SIRT1 or p53 expression, or in cells incubated with Sirtinol (100 μg/ml for 24 h). These results suggested that both SIRT1 and p53 functions play a critical role in transcription inhibition of the core mTERT promoter.

### ΔNp63α modulates the RNA splicing of mTERT

We have previously shown that ΔNp63α is implicated in both transcriptional regulation and post-transcriptional processing/splicing of downstream target genes [31,55].   We previously reported that the ΔNp63α protein physically associated with ABBP1, one of the key components of RNA processing molecular machinery [55]. We found that the ΔNp63α ABBP1 protein complexes contributed into the fibroblast growth factor receptor 2 receptor RNA splicing leading to epithelial-mesenchymal transition [55].  Here we report that these ΔNp63α ABBP1 protein complexes were also involved in the post-transcriptional regulation of mTERT.

Telomerase is a reverse transcriptase that adds telomeric repeats d(TTAGGG)n to chromosomal ends [56].   In most normal somatic cells, telomerase is repressed and telomeres progressively shorten, leading to limited proliferative lifespan [2,56].  Telomerase reactivation is associated with cellular immortalization and is a frequent event during tumorigenesis [2,11,13]. Structurally telomerase is a ribonucleoprotein complex that consists of two essential components, TERT and a template RNA, TR [56].  Telomerase ribonucleoprotein complex plays a critical role in ageing, tumorigenesis, immortalization and "stemness" phenotype [2,11,13,20,23,24,57].  A number of reports pointed-out that a major control mechanism underlying the telomerase function lies at the level of transcription and alternative splicing of TERT [18,42,48,49,51,53,59-62].

**Figure 4.**
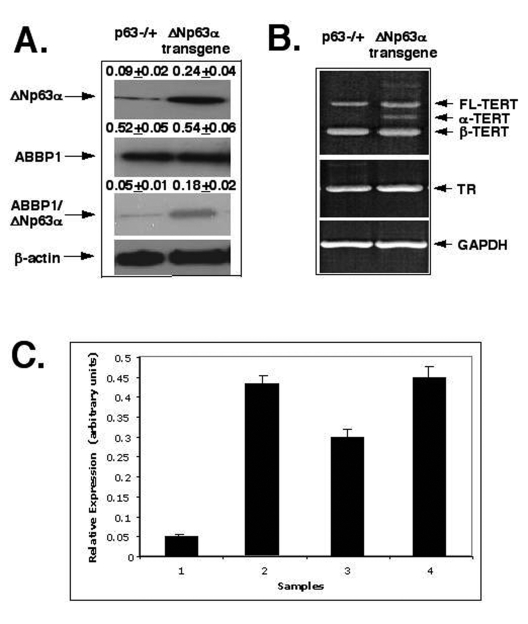
ΔNp63α increases levels of the mTERT-spliced isoforms via protein interaction with ABBP1. Mouse epidermal keratinocytes (2x10^6^ cells) expressing heterozygous *p63-/+* and *ΔNp63α* transgene. **(A)** Cells were tested for the levels of ΔNp63αand ABBP1 by immunoblotting and ABBP1ΔNp63αprotein complexes using immunoprecipitation (IP) with an antibody to ABBP1 followed by immunoblotting with an antibody to ΔNp63α. As a control, the protein level of β-actin was monitored. **(B)** Cells were examined for the expression of the mTERT and mTR transcripts using RT-PCR. GAPDH was used in RT-PCR assay, as a control. **(C)** The relative expression of TERT and TR was quantitatively analyzed and plotted as bars using the Microsoft Excel software. All of the data (mean +SD) were from at least three independent experiments.  Samples: cells from *p63-/+*    mice, 1- TERT/GAPDH ratio; 2- TR/GAPDH ratio; cells from the *ΔNp63α*transgenic mice, 3- TERT/GAPDH ratio; 4- TR/GAPDH ratio. PCR experiments with the 2164/ 2620 set of primers generated three products that represent the full-length TERT transcript (457 bp), the α-splice transcript (421 bp), and the β-splice transcript (275 bp). Sequence analysis revealed that the longer transcripts were full-length one and the shorter transcripts were α and β- spliced messages of mTERT.

We thus investigated whether the overexpression of the ΔNp63α protein would affect the RNA splicing of mTERT and mTR in mouse epidermal cells.  First, we observed that the ΔNp63α overexpression led to an increasing level of the ΔNp63α ABBP1 protein complexes (by 3-4-fold, Fig. 4A). Second, we found that the ΔNp63α overexpression failed to affect expression of RNA component of telomerase complex (mTR) as shown in Figure 4B (middle panel).  And finally, we found that the ΔNp63α overexpression dramatically induced the levels of α-splice isoform (by 5-6-fold) and β-splice isoform (by 1.5-2-fold) of mTERT (Fig. 4B, upper panel and Fig. 4C), while levels of the full-length isoform of mTERT remained unchanged in mouse epidermal keratinocytes from the *ΔNp63α* transgenic mice compared to such levels found in cells from *p63-/*+ mice (Fig. 4B, upper panel).  As previously reported, these variants were not equal in their ability to generate an active TERT complex [63-66].   Telomerase activity is only provided by the full-length TERT [63-66].  The smaller splice variants (α and β) are inactive and may act as dominant-negative inhibitors for telomerase activity [63-66].   The α-splice isoform lacks a 12-residue region of the conserved reverse transcriptase motif A (in-frame deletion), and the β-splice is missing a 182 bp-region resulting in a non-sense mutation leading to premature stop codon, truncating the protein before the conserved reverse transcriptase motifs B, C, and D [63-66].

### ΔNp63α modulates telomerase activity and increases cellular senescence

**Figure 5.**
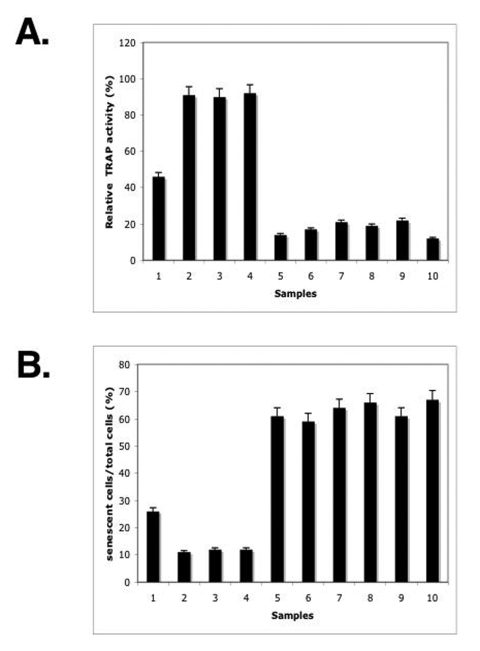
ΔNp63α overexpression modulated the overall telomerase activity and induced a S-β-gal activity. The mouse keratinocytes from the *p63-/+*    mice (samples 1, 3, 5, 7, 9) and *ΔNp63α*transgenic mice (samples 2, 4, 6, 8, 10) were treated with the control media (samples 1 and 2) or Sirtinol (100 μg/ml for 24h, samples 3 and 4) or transfected for 72h with shRNA against SIRT1 (samples 5 and 6), p53 (samples 7 and 8) and Sp1 (samples 9 and 10). **(A)** Telomerase activity. Telomerase activity was determined by the TRAP assay using 1 μg of protein extract obtained from 2x10^5^ cells. Quantitative analysis was done using Molecular Dynamics densitometer and ImageQuant software. The intensity of the positive control lane was taken as 100%. The experiment was repeated three times, and error bars represent mean ± S.D. **(B)** S-β-gal activity. The S-β-gal activitywas measured using a senescence kit.

To further examine the effect of the ΔNp63α overexpression on the overall telomerase activity, we obtained the mouse epidermal keratinocytes from the *ΔNp63α* transgenic mice and *p63-*/- heterozygous mice. Cells were transfected with shRNA against SIRT1, p53 or Sp1 for 72 h or treated with Sirtinol for 24 h. Resulting cells were tested for telomerase activity using the TRAP assay, as described elsewhere [63-66].  We first found that the level of telomerase activity in keratinocytes from *p63+/*- mice is significantly greater than in cells from the *ΔNp63α* transgenic mice (Fig. 5A, samples 1 and 6). Second, we observed that the treatment with either SIRT1 inhibitor or shRNA against SIRT or p53 led to an increase in telomerase activity in keratinocytes from *p63+/*- mice (Fig. 5A, samples 2-4), while no significant changes were seen in the *ΔNp63α* transgenic mice (Fig. 5A, samples 7-9). Third, we showed that the Sp1 shRNA dramatically decreased the telomerase activity in both mouse models (Fig. 5A, samples 5 and 10).

The inhibition of endogenous telomerase activity resulting in telomere shortening was shown to lead to a replicative senescence [11-22].  We previously showed that the forced overexpression of ΔNp63α led to an increase in replicative senescence of human squamous cell carcinoma cells that were distinguished by the presence of a biomarker - senescence-associated β-galactosidase (S-β-gal) as described [29]. Senescent cells show a series of morphological and physiological alterations including a flat and enlarged morphology, an increase in acidic S-β-gal activity, chromatin condensation, and changes in gene expression pattern. Here we observed that the level of the S-β-gal activity in keratinocytes from *p63*+/- mice is significantly lower than in cells from the *ΔNp63α* transgenic mice (Fig. 5B, samples 1 and 6).  Then, we showed that the treatment with either Sirtinol or SIRT shRNA or p53 shRNA led to a decrease in the S-β-gal activity in keratinocytes from *p63*-/+ mice (Fig. 5B, samples 2-4). Finally, we found that the Sp1 shRNA dramatically increased the S-β-gal activity in *p63-*/+ mice (Fig. 5B, sample 5).  In the same time, epidermal keratinocytes obtained from the *ΔNp63α* transgenic mice failed to display significant changes in the S-β-gal activity under above-mentioned experimental conditions (Fig. 5B, samples 7-10).

**Figure 6.**
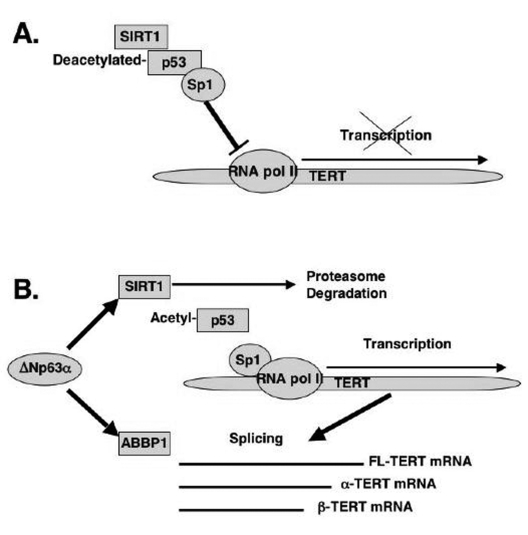
Schematic representation of regulation of TERT transcription and splicing by ΔNp63α. (A) mTERT transcription. (B) mTERT splicing.

## Discussion

Normal somatic cells undergo a limited number of divisions before entering an irreversible growth-arrest state, a replicative cellular senescence, providing a barrier against the unlimited proliferation and formation of cancer [1-7]. The molecular mechanism underlying the replicative senescence involves the telomere shortening due to the inability to renew the telomere length by a telomerase enzymatic complex [56]. Telomeres are specific DNA-protein complexes present at the ends of linear chromosomes, which protect the latter from degradation and fusion/recombination [56]. Telomeric DNA is synthesized by a multisubunit enzymatic complex, telomerase, consisting of the telomerase reverse transcriptase (TERT), an RNA component (TR) acting as a template, and other associated proteins [56].  Replicative senescence can be overcome by overexpression of the catalytic subunit of telomerase-reverse transcriptase (TERT) as previously reported [59]. A growing number of reports showed that various treatments could induce premature senescent phenotype through regulation of TERT [3,5,8,10,15,25,52,57]. They include various types of DNA damage, overexpression of oncogenes or mitogenic signals, and changes affecting chromatin structure [3,5,8,10,15,25,52,57].   Replicative senescence is likely to play a role in ageing of highly proliferative tissues such as skin, endothelium and lymphoid tissues [18,60,62,64].

Telomerase activity closely correlates with the expression of TERT, which could potentially be regulated at the transcriptional (promoter) and post-transcriptional (splicing) levels [2].  The TERT promoter activity is usually regulated by a variety of transcription factors (AP-1, c-Myc, Sp1, Sp3, NF-kB, Ets, and the estrogen receptor), and by chromatin remodeling and epigenetic methylation mechanisms [3,5,8,10,15,25,48,52,57].   Several variants of TERT are also generated by RNA splicing within the reverse transcriptase region and the C-terminal part of the TERT gene and shown to function as endogenous dominant-negative regulators/inhibitors of telomerase activity [63-66].  Cells from skin cancers (melanomas) were shown to produce the complete TERT mRNA along with one or more alternatively spliced transcripts [62].  Depending of ratio between the full-length TERT and spliced TERT isoforms, melanoma cells were characterized as positive and negative for telomerase activity [48,62].  The high abundance of spliced TERT isoforms dramatically inhibited the overall telomerase activity [62].

We previously showed that the premature ageing in the *p53+/m* and *ΔNp63α* mice was accompanied by increased ΔNp63α expression leading to induced cellular senescence that was rescued by SIRT1 suggesting that ΔNp63α levels may affect ageing through regulation of SIRT1 [29].  Modulation of p63 function through genetic knockdown/RNA silencing [25, 26] or by dominant-negative inhibitor, ΔNp63α [29], could lead to a premature ageing phenotype, however implicating SIRT1 regulation into the molecular mechanism underlying the organismal ageing process [29,32-40].

In the current report, we showed that from the first hand, ΔNp63α induced the mTERT promoter activation through the down regulation of the SIRT1 protein levels, inactivation of p53 deacetylation, decrease of the p53/Sp1 protein-protein complexes, and the overall induction of mTERT transcription regulation (Fig. 6A). From the other hand, by a forming of protein-protein complexes with the ABBP1-derived RNA processing/splicing complex (Fig. 6B), ΔNp63α induced the mTERT RNA splicing leading to an increasing expression of spliced mTERT isoforms playing a role of dominant-negative inhibitors of mTERT activity and therefore decreasing the levels of TERT activity in mouse epidermal keratinocytes overexpressing the ΔNp63α protein. The overall effect of the ΔNp63α overexpression resulted in decrease in telomerase activity and increase in replicative senescence observed in mouse keratinocytes.  This dual molecular mechanism of telomerase regulation might underline the previously shown effect of p63 (ΔNp63α) on premature ageing phenotype observed in mice overexpressing the ΔNp63α protein.

## Methods

Antibodies and reagents. We used a rabbit polyclonal antibody to ABBP1 (raised against the C-terminal peptide SQRRGGHQNNYKPY by Affinity Bio-Reagents), a goat polyclonal antibody to ΔNp63 (N-16, sc-8609), and a mouse monoclonal antibody to p63 (4A4, sc-8431, both from Santa Cruz Biotechnology), a mouse monoclonal antibody to SIRT1 (#07-131, Upstate Cell Signaling Solutions), and a rabbit polyclonal antibody to mouse Sp1 (#S9809, Sigma) or a rabbit polyclonal ChIP-grade antibody against Sp1 (Upstate Biotechnology). We also used an agarose-conjugated p53 monoclonal antibody (Ab-6; Oncogene Research Products), and anti-acetylated-Lys382 p53 antibody (Cell Signaling Technology). We also used the SIRT1 inhibitor, Sirtinol (#566320-5MG), and a 26S proteasome inhibitor, MG-132 (474791-1MG) that were purchased from Calbiochem.

Preparation of mouse keratinocytes. *P63-/+*mice harboring the *p63Brdm2* allele (obtained from Jackson Laboratories) and ΔNp63α transgenic mice (generated in our laboratory) were used [29,45] according to the regulations of the Johns Hopkins University Animal Care and Use Committee (JHUACUC). Primary keratinocytes were isolated from 3-4 day-old newborn pups by a trypsinization [46,47].    Cells were plated at 3 x 10^6^ cells per 60-mm dish in chelex-treated low-calcium EMEM medium, BioWhittaker) supplemented with 8% fetal bovine serum and 0.05 mM calcium) and grown at 37^o^C with 5% CO_2_.  Total lysates were obtained from cells flash-frozen in liquid N_2_ and transferred into a buffer A as described [55]. The samples were homogenized on ice and centrifuged at 15,000 x g for 20 min at 4°C.  The supernatants were separated on 12.5% SDS-PAGE gels and probed with indicated antibodies.  Immunoblotting and immuno-precipitation was performed as described [55].

TERT promoter luciferase assay. The mouse TERT promoter  region  encompassing  Sp1 binding element(-347 to +1) was kindly obtained from Drs. Charles Giardina and Rashimi R. Joshi, University of Connecticut, Sparks, CT) as previously described [53,54].  For the luciferase assay, mouse keratinocytes (1.0 x 105) were transfected with the pGL3-347-Luc plasmid (0.5 μg) or the pGL3 control plasmid (0.5 μg) by using FuGENE6 transfection reagent (Roche Diagnostics). 3 ng of the pRL-SV40 (Promega) was used as a normalization control. Cell lysates were obtained and measurements were performed by using the Dual Luciferase reporter assay system (Promega) and a BioOrbit 1251 luminometer.  The activity of each TERT promoter fragment was expressed as a relative value. All of the data (mean +SD) were from at least three independent experiments.

Small hairpin RNA (shRNA), design and manipulation. ShRNA for mouse SIRT1 (#TR505485), Sp1 (#TR502115) and p53 (#TG500002) and scrambled shRNA were purchased from Origene Technologies and used according to the manufacturer's recommendations. Control and experimental shRNA (200 pmol/six-well plate) were transiently introduced into mouse keratinocytes with aid of TurboFectin 8.0 (Origene Technologies), and 72 h later, total lysates were used for immunoblotting.

Telomerase activity assay. For telomerase activity detection, we used the PCR-mediated telomere repeat amplification protocol (TRAP) as previously described [63-66]. As a negative control, cell extract was substituted for lysis buffer.  Two μl of cell lysate (protein concentration 0.5 μg/μl) were used per assay.  The PCR products were run on a 10% polyacrylamide gel, stained with SYBR Green (BioWhittaker Molecular Applications), and detected using the Typhoon system (Molecular Dynamics). For quantitative analysis, the ImageQuant version 5.2 software (Molecular Dynamics) was used. The area of integration of all peaks was normalized to the signal from the internal standard, then, after background subtraction, expressed relative to the positive control signal (100 cell equivalent) that was run with each experiment. The comparison of mean values between the different groups was evaluated by ANOVA with Fisher's LSD test.

RT-PCR assay. Total RNA was isolated from cells using Trizol reagent (Invitrogen). One μg of total RNA was used to generate a cDNA from each sample using one-step RT-PCR kit (Qiagen) and custom primers. mTR expression was monitored using the following primers: sense (mTR, +1) 5'-CGTAATACGACTCAC TATAGGGT-3' and antisense (mTR, +451), 5'-GCATGTGTGAGCCGAGTCCT-3' as described elsewhere [56].  The mTERT spliced variants were detected with the following primers [56]: sense, 5'-GCCTGA GCTGTACTTTGTCAA-3', and antisense, 5'-CGCA AACAGCTTGTTCTCCATGTC-3'. As a positive control we amplified the glyceraldehyde 3-phosphate dehydrogenase (GAPDH) was amplified with the following primers: sense, 5'-ACCACAGTCCATGCCA TCAC-3' and antisense, 5'-TCCACCACCCTGTTGCT GTA-3'.  PCR products were separated by 2% agarose or in 4-20% gradient non-denatured PAG electrophoresis and were visualized with ethidium bromide. All RT-PCR data was analyzed digitally by Kodak 1D 3.5 software. The net intensity of RT-PCR bands for the full-length mTERT and mTR were measured and normalized by net intensity of GAPDH bands.

Chromatin immunoprecipitation (ChIP). ChIP assays were performed using the antibody against mouse Sp1 (Upstate Biotechnology) or rabbit immunoglobulins as negative controls (Sigma) and ChIP assay kit (Upstate) on primary mouse keratinocytes as previously described [53,54].  The proteins bound to DNA were cross-linked using 1% formaldehyde for 10 min at 37°C and the protein-DNA complexes were precipitated with a primary antibody against Sp1. After reversal of the cross-links and DNA recovery, the latter was used as a template to amplify the mTERT-derived Sp1 promoter region using the following primers: sense, 5'-CTCA CTGTCTGTGCAACCACAGCAGCTG-3' (position-363), and antisense, 5'-AGAGCACCGCGGGGCAA CGAGGAGCGCG-3' (position +143) producing a 506 bp PCR product. The PCR products were run on 2% agarose gels and visualized by ethidium bromide.

Senescence-associated β -galactosidase (S- β -gal) activity. Mouse keratinocytes were transfected with shRNA for 72 h or treated with Sirtinol for 24 h prior to assaying. The S-β -gal activity was measured using a senescence kit (Cell Signaling). Briefly, the cells were fixed with 3% formaldehyde solution [29]. The cells were then washed and incubated with staining solution (1 mg/l, 5-bromo-4-chloro-3-indolyl-β-D-galactopyranoside (X-gal), 40 mM citric acid/sodium phosphate buffer, pH 6.0, 5 mM ferrocyanide, 5 mM ferricyanide, 150 mM NaCl, and 2 mM MgCl_2_) for 12-18 h to visualize S-β-gal activity as described [52].  Data were plotted as ratio of senescent cells over total cells using Microsoft Excel software. All of the data (mean +SD) were from at least three independent experiments.
